# Cell-cell interaction in the pathogenesis of inherited retinal diseases

**DOI:** 10.3389/fcell.2024.1332944

**Published:** 2024-03-04

**Authors:** Xue Du, Anna G. Butler, Holly Y. Chen

**Affiliations:** Department of Cell, Developmental and Integrative Biology, Heersink School of Medicine, University of Alabama at Birmingham, Birmingham, AL, United States

**Keywords:** cell-cell interaction, inherited retinal disease, photoreceptor, Müller glia, retinal pigment epithelium

## Abstract

The retina is part of the central nervous system specialized for vision. Inherited retinal diseases (IRD) are a group of clinically and genetically heterogenous disorders that lead to progressive vision impairment or blindness. Although each disorder is rare, IRD accumulatively cause blindness in up to 5.5 million individuals worldwide. Currently, the pathophysiological mechanisms of IRD are not fully understood and there are limited treatment options available. Most IRD are caused by degeneration of light-sensitive photoreceptors. Genetic mutations that abrogate the structure and/or function of photoreceptors lead to visual impairment followed by blindness caused by loss of photoreceptors. In healthy retina, photoreceptors structurally and functionally interact with retinal pigment epithelium (RPE) and Müller glia (MG) to maintain retinal homeostasis. Multiple IRD with photoreceptor degeneration as a major phenotype are caused by mutations of RPE- and/or MG-associated genes. Recent studies also reveal compromised MG and RPE caused by mutations in ubiquitously expressed ciliary genes. Therefore, photoreceptor degeneration could be a direct consequence of gene mutations and/or could be secondary to the dysfunction of their interaction partners in the retina. This review summarizes the mechanisms of photoreceptor-RPE/MG interaction in supporting retinal functions and discusses how the disruption of these processes could lead to photoreceptor degeneration, with an aim to provide a unique perspective of IRD pathogenesis and treatment paradigm. We will first describe the biology of retina and IRD and then discuss the interaction between photoreceptors and MG/RPE as well as their implications in disease pathogenesis. Finally, we will summarize the recent advances in IRD therapeutics targeting MG and/or RPE.

## 1 Introduction

Among the five senses of humans (vision, taste, touch, smell, hearing), vision is considered as the most important one for most people (Hutmacher, 2019). Approximately a hundred years ago, research on perception and perceptual memory was mainly focused on the vision, through which we perceive most of information (Ripley and Politzer, 2010; Hutmacher, 2019). Vision starts with light entering the eye, being filtered through the cornea, and focused onto the retina by the lens. The retina is a multi-layered structure specialized for vision. The light-sensitive photoreceptors in the retina capture photons and convert them into electrical impulses, which are integrated and processed by interneurons, and transmitted to the lateral geniculate nucleus, pretectal nuclei, and superior colliculus in the brain by retinal ganglion cells (Smith and Czyz, 2023).

Up to 75% of people consider losing the sense of vision the scariest compared to the other four senses (Hutmacher, 2019). Vision loss stems from the inability of the retina to detect the light and/or transmit visual signals to the brain (Wright et al., 2010; Veleri et al., 2015; Chen et al., 2021b). Amongst the plethora of blinding disorders, inherited retinal diseases (IRD) have particular significance. IRD are a group of clinically and genetically heterogeneous disorders characterized by progressive vision impairment or loss. Although individually rare, IRD have an accumulative prevalence of up to 5.5 million cases globally (Ben-Yosef, 2022). As a major cause of childhood blindness (John et al., 2022), IRD frequently destine children to a lifetime of severe vision impairment and/or blindness and cause a considerable burden on family and societies (Leroy et al., 2021). In 2019, the total costs spent on IRD were estimated between $13.4 and $31.8 billion in the United States and between $1.6 and $6.7 billion in Canada (Gong et al., 2021). Currently, there is only one FDA-approved gene therapy drug voretigene neparvovec (Luxturna^®^) to treat IRD caused by *RPE65* mutations (Pierce and Bennett, 2015), and the long-term effect seems variable (Gardiner et al., 2020; Wang et al., 2020; Leroy et al., 2022). In addition, with over 280 disease-causing genes of IRD (RetNet, https://sph.uth.edu/retnet/), the development of individualized gene therapy protocols would not be an optimal option particularly for an individually rare disease (Tambuyzer et al., 2020). Therefore, gene-agnostic paradigms are being developed as a more desirable therapeutic approach (Scholl et al., 2016) and a comprehensive understanding of the cellular and molecular mechanisms of IRD is a premise for designing effective and long-lasting therapeutics.

A majority of IRD are due to dysfunction and/or degeneration of the light-sensitive photoreceptors (Wright et al., 2010; Verbakel et al., 2018), with disruption of photoreceptor outer segment biogenesis/function, phototransduction, synapses, metabolism the most frequent causes (Veleri et al., 2015; Zelinger and Swaroop, 2018). We note that development and homeostasis of photoreceptors heavily rely on the interaction with the retinal pigment epithelium (RPE) and Müller glia (MG). Consistently, an increasing number of studies suggest that pathologies of RPE or MG may compromise photoreceptor survival (Wright et al., 2010; Mysore et al., 2014; Pellissier et al., 2015; Veleri et al., 2015; Duncan et al., 2018; Amato et al., 2021; Chen et al., 2023a). Therefore, degeneration of photoreceptors in IRD could be a direct consequence of genetic mutations and/or secondary to compromised RPE/MG. This review aims to summarize our current understanding in the interaction among these cell types and explore how dysfunction of one cell type could compromise the other one, a process that is particularly important in designing long-lasting and efficacious therapeutics. As some of these mechanisms could be common among various mutations, a comprehensive understanding of the mechanisms underlying the cell-cell interaction in the outer retina should hold the promise to identify therapeutic targets for gene-agonistic therapies.

## 2 Retinal structure and function

**FIGURE 1:**
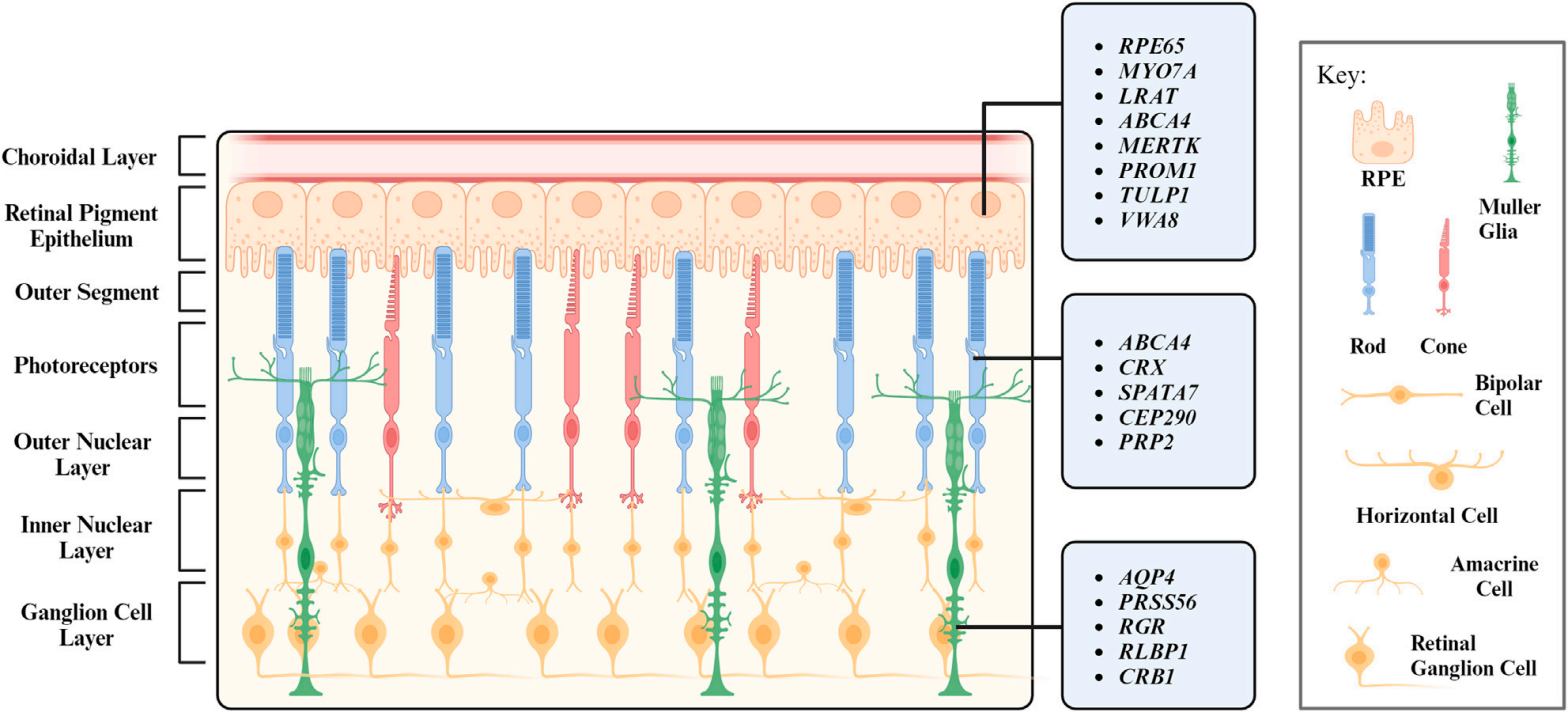
Layers of the retina and their respective cell types. Genes associated with inherited retinal degeneration expressed in retinal pigment epithelium (RPE), photoreceptors (PR), and Müller Glia (MG) are listed.

When light strikes the eye, the cornea and the lens bend and invert the light to focus it on the retina, a receptive inner layer lining the posterior part of the eyes (Masland, 2012; Erskine and Herrera, 2014). The retina is comprised of neurons (photoreceptors, bipolar cells, horizontal cells, amacrine cells, and retinal ganglion cells) and glial cells (MG) forming three distinct cellular layers [the outer nuclear layer (ONL), inner nuclear layer (INL), and ganglion cell layer (GCL)] and two plexiform layers (the outer plexiform layer and inner plexiform layer) enriched in cellular processes and synapses (Figure 1) (Hoon et al., 2014).

Every retinal cell type has its unique and vital role in maintaining the retinal function. Photoreceptors are light-sensitive neurons located at the ONL and can be further categorized into rod and cone photoreceptors based on their morphology and function. Rod photoreceptors were historically considered to function under dim light for night vision. Recent studies reveal they also support cone survival and function (Leveillard et al., 2004; Pahlberg et al., 2017; Tikidji-Hamburyan et al., 2017). Cone photoreceptors operate at bright illumination conditions and are responsible for color and high acuity vision as well as non-image forming responses (Gooley et al., 2010; Lall et al., 2010; Molday and Moritz, 2015; Walmsley et al., 2015). Retinal interneuron bipolar, horizontal, and amacrine cells are mainly located at the INL. Bipolar cells process visual signals from photoreceptors and transmit them to retinal ganglion cells, a process modulated by horizontal cells and amacrine cells with both excitatory and inhibitory properties (Diamond, 2017). Visual signals are transmitted to the brain via the optic nerves, which are axons of the retinal ganglion cells (Mead and Tomarev, 2016).

The structure and function of the retina is maintained by MG, which are the most abundant glial cells in the retina. They play a crucial role in maintaining the homeostasis of retina and provide structural, metabolic, and functional support to retinal neurons (Reichenbach and Bringmann, 2013; Tworig and Feller, 2021). RPE is another type of supporting cell for the retina. Located juxtaposed to photoreceptors (Figure 1), RPE form the outer retina-blood barrier, with their apical and basal side interfacing with photoreceptors and the Bruch’s membrane, respectively (Lakkaraju et al., 2020). RPE tightly interact with photoreceptors to coordinate metabolism and visual cycle (Palczewski and Kiser, 2020; Hurley, 2021; Nolan et al., 2021). We will further discuss the interaction between photoreceptors and their interaction partners MG and RPE in the following sections.

## 3 Genetics and biology of inherited retinal degenerative diseases

The genesis and health of the retina are maintained by numerous structural and functional components. Mutations in genes encoding for these components could lead to progressive, visually debilitating diseases collectively termed as IRD. The discovery of the first two IRD-causing genes (*RHO* and *CHM*) can be traced back to 1990 (Cremers et al., 1990; Dryja et al., 1990). With the advances in sequencing technologies and genetic mapping, more than 300 IRD-associated loci are mapped and over 280 disease-causing genes with diverse roles in the retina have been identified (RetNet; https://web.sph.uth.edu/RetNet/).

IRD are highly heterogeneous both genetically and phenotypically. They can be monogenic, digenic, or even more complex, and inherited as autosomal recessive, autosomal dominant, or X-linked. Recently, a comprehensive analysis on 1243 proband-parent trios in 22 subgroups of inherited eye disorders by targeted exome sequencing reveals *de novo* mutations contributes to approximately 7% of pathogenicity (Li et al., 2023). *De novo* mutations could arise from patients with a simplex disease and cause autosomal dominant phenotypes, such as in the case of the Arg677Ter mutation of *RP1* (Schwartz et al., 2003). The presence of *de novo* mutations supports the hypothesis of mutational hotspots (Schwartz et al., 2003; Daiger et al., 2007). Besides RP1, a recent study on a large cohort indicates the high prevalence of *ABCA4* and *USH2A* mutations (Karali et al., 2022). The pleiotropic effect of numerous IRD-causing genes, in which mutations in one gene could cause diverse phenotypes, adds more complexity to the clinical manifestations (den Hollander et al., 2010; Valente et al., 2006; Siemiatkowska et al., 2014; Daiger et al., 2007). More than 50 major types of IRD have been documented globally, with retinitis pigmentosa (RP), Leber congenital amaurosis (LCA), Stargardt disease as the most common forms (Duncan et al., 2018; Schneider et al., 2022). RP, which is the most prevalent IRD and contributes to approximately half of the IRD cases (Daiger et al., 2013), is characterized by initial rod photoreceptor degeneration followed by gradual loss of cone cells (Verbakel et al., 2018). LCA is a group of severe retinal dystrophy and the leading cause of inherited childhood blindness (Kumaran et al., 2017). Visual impairment or blindness caused by dysfunction or degeneration of photoreceptors is congenital or manifested within the first few months after birth in LCA patients (Cideciyan and Jacobson, 2019). Different from RP and LCA, in which compromised peripheral vision caused by dysfunctional rods is the earliest and most common phenotype (Shintani et al., 2009; Chacon-Camacho and Zenteno, 2015), Stargardt disease is the most prevalent inherited macular dystrophy characterized by gradual loss of central vision (Gelisken and De Laey, 1985; Weleber, 1994). Most Stargardt patients experience significant reduction in visual acuity in their first or second decade of life, which is associated with loss of photoreceptors and/or RPE (Molday, 2015).

While dysfunction or death of photoreceptors is a common phenotype shared by almost every type of IRD, disease-causing genes encode for proteins involved in diverse cell types, signaling pathways, and cellular functions. Notably, numerous IRD with photoreceptor degeneration are caused by mutations in genes associated with RPE and MG structure/function (Table 1, 2). Therefore, photoreceptor degeneration in IRD could be a direct cause of genetic mutations and/or secondary to pathologies of its interaction partner such as MG and RPE. In favor of the latter, therapeutic approaches targeting MG and RPE show promising outcome in maintaining photoreceptor survival and function in preclinical models and clinical trials (Cideciyan et al., 2009; Jacobson et al., 2012; LaVail et al., 2016; Buck et al., 2021). In the following sections, we will focus on the function of RPE and MG as well as their interaction with photoreceptors, with an aim to unravel their potential role in IRD pathogenesis.

**TABLE 1:** Retinal pigment epithelium (RPE) genes associated with inherited retinal degeneration^a^.

OMIM#	Gene	Protein	Cell type	Function	Phenotype^b^
604210	CRB1	Crumbs homolog 1	RPE	Maintenance of retinal cell junction and retinal polarity	Leber congenital amaurosis (LCA) 8; Retinitis Pigmentosa (RP) 12; Pigmented paravenous chorioretinal atrophy
300757	RP2	RP2 activator of ARL3 GTPase	Photoreceptor; RPE (Schwarz et al., 2015)	Activating GTPase in tubulin cellular pathway; ciliary traffic	RP 2
609868	SPATA7	Spermatogenesis-associated protein 7	Photoreceptor; RPE (Sengillo et al., 2018)	Manufacturing of RPGRIP1 protein complex in the cilia	RP 94; LCA 3
604863	LRAT	Lecithin retinol acyltransferase	RPE	Processing visual cycle molecules	Juvenile RP; LCA 14; Retinal dystrophy
601691	ABCA4	ATP-binding cassette superfamily transmembrane protein	Photoreceptor; RPE (Lenis et al., 2018)	Removing the toxic byproduct of the visual cycle	RP 19; Stargardt disease 1; Macular degeneration; Fundus flavimaculatus
604705	MERTK	MER tyrosine kinase proto-oncogene	RPE	Modulating the RPE phagocytosis pathway	RP 38
600342	RGR	RPE-retinal G protein-coupled receptor	RPE; Müller glia (MG)	Facilitating the isomerization of 11-*cis*-retinal	RP 44
180069	RPE65	Retinoid isomerohydrolase	RPE	Converting all-*trans*-retinyl ester to 11-*cis*-retinol in the visual cycle	RP 20 and 87; LCA 2
607854	BEST1	Bestrophin 1	RPE; MG	Formation and function of chloride ion channels	RP 50; Vitreoretinochoroi-dopathy; Bestrophinopathy; Macular dystrophy
606151	BBS2	Bardet-Biedl syndrome 2 (part of the BBSome)	Photoreceptor; RPE	Ciliogenesis	RP 74; Bardet-Biedl syndrome 2
607292	SEMA4A	Semaphorin 4A	RPE; Retinal ganglion cells; Amacrine cells	Retinal development	RP 35; Cone-rod dystrophy 10
608132	TTC8	Tetratricopeptide repeat domain-containing protein 8 (part of the BBSome)	RPE	Ciliogenesis	RP 51; Bardet-Biedl syndrome 8
607056	IMPG2	Interphotoreceptor matrix proteoglycan 2	Photoreceptor; RPE	A component of the extracellular matrix between RPE and photoreceptors	RP 56; Macular dystrophy
300170	OFD1	OFD1 centriole and centriolar satellite protein	RPE	A modulator of centriole length	RP 23, Joubert Syndrome 10
608845	ARL6	ADP-ribosylation factor-like GTPase 6	RPE	Binding the BBS complex to the cilia membrane	RP 55; Bardet-Bledl syndrome 1 and 3
617539	CLCC1	Chloride channel CLIC-like 1	RPE; Photoreceptor (Li et al., 2018)	Chloride channel activity and transport on the cellular and mitochondria-associated ER membrane	RP 32
609507	TOPORS	Topoisomerase I-binding arginine/serine-rich protein	RPE; photoreceptor; retinal ganglion cells	Ubiquitin-protein E3 ligase	RP 31
617509	VWA8	von Willebrand factor A domain containing 8	RPE	Potential functions in mitochondria, autophagy, apoptosis, and retinal development	RP 97
602280	TULP1	TUB-like Protein 1	Photoreceptor; RPE; MG; Retinal ganglion cells (Palfi et al., 2020)	Protein trafficking; Ligand in the MERTK pathway for RPE phagocytosis (Palfi et al., 2020)	RP 14; LCA 15
146690	IMPDH1	IMP Dehydrogenase 1	Photoreceptor; RPE (Kennan et al., 2003)	Precursor of GMP and guanin; Modulating the formation of IMP	RP 10; LCA 11
604365	PROM1	PROMININ	Photoreceptor; RPE	Outer segment disc morphogenesis	Cone-rod dystrophy; Macular dystrophy; RP 41; Stargardt disease 4
276903	MYO7A	Myosin VIIA	RPE; Photoreceptor	Transport of melanosome and phagosome	Usher syndrome

^a^ Unless otherwise specified, this table summarizes information of RPE-associated genes in OMIM, Online Mendelian Inheritance in Man (https://www.omim.org/).

^b^ We note that multiple genes in inherited retinal degeneration are associated with syndromic disorders with multi-organ pathology. Only phenotypes associated with the retina are listed in the table.

**TABLE 2:** Müller glia (MG) genes associated with inherited retinal degeneration^a^.

OMIM#	Gene	Protein	Cell type	Function	Phenotype^b^
600308	AQP4	Aquaporin 4	MG (Li et al., 2002)	Modulating water homeostasis in the retina	Inherited retinal dystrophy (Lassiale et al., 2016)
613858	PRSS56	Serine protease 56	MG (Paylakhi et al., 2018)	Modulating ocular axial growth	Microphthalmia, isolated 6
600342	RGR	Retinal G protein-coupled receptor	Retinal pigment epithelium (RPE); MG (Jiang et al., 1993)	Converting all-*trans* retinal to 11-*cis* retinal	Retinitis pigmentosa 44
180090	RLBP1	Retinaldehyde binding protein 1	RPE and MG (Maw et al., 1997)	Visual cycle	Bothnia retinal dystrophy; Fundus albipunctatus; Newfoundland rod-cone dystrophy; Retinitis punctata albescenes
604210	CRB1	Crumbs cell polarity complex component 1	Photoreceptor; MG (Pellissier et al., 2014)	Cellular polarity	Leber congenital amaurosis 8; Retinitis pigmentosa 12; Pigmented paravenous chorioretinal atrophy

^a^ Unless otherwise specified, this table summarizes information of MG-associated genes in OMIM, Online Mendelian Inheritance in Man (https://www.omim.org/).

^b^ We note that multiple genes in inherited retinal degeneration are associated with syndromic disorders with multi-organ pathology. Only phenotypes associated with the retina are listed in the table.

## 4 Photoreceptor-RPE interaction in IRD

**FIGURE 2:**
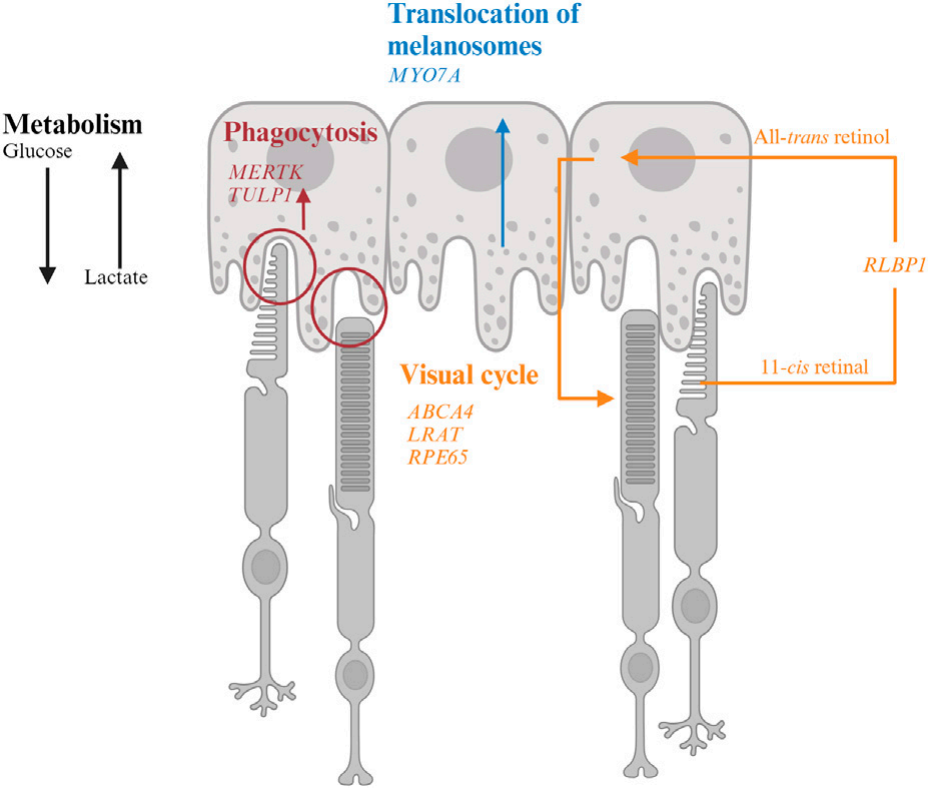
Interaction of retinal pigment epithelium (RPE) and photoreceptor and genes associated with inherited retinal degeneration. RPE harbor melanosomes that absorb light. The distribution of melanosomes is modulated by MYO7A. MERTK and TULP1 facilitate phagocytosis of photoreceptor outer segments and their mutations could disrupt the metabolic homeostasis between photoreceptors and RPE. *LRAT*, *RPE65*, and *ABCA4* encode for key molecules in visual processing. The transport of 11-*cis* retinal from RPE to photoreceptors and all-*trans* retinol from photoreceptors to RPE are facilitated by CRALBP encoded by *RLBP1*.

RPE form a monolayer between the outer retina and the choroidal layer (Figure 1). The basolateral side of RPE faces the Bruch’s membrane, an elastin- and collagen-rich extracellular matrix between the RPE and the fenestrated choroidal capillaries of the eye, and the apical side is juxtaposed to photoreceptors (Strauss, 2005; Booij et al., 2010). The tight junction of RPE enables the layer to act as a semipermeable membrane to provide structural and functional support for the retina. Due to the close relationship between photoreceptors and RPE, mutations in RPE genes are often associated with photoreceptor degeneration in IRD (Table 1). In this section, we will discuss how mutations in RPE-associated disease-causing genes abrogate RPE function and lead to photoreceptor degeneration in IRD (Figure 2).

### 4.1 Absorption of light and reduction of oxidative stress

While light is essential for vision, exposure to bright illumination could cause permanent photic damage to the retina (Youssef et al., 2011). Human RPE contain three types of pigment granules at various stages, which absorb and filter approximately 60% of light with various wavelengths to protect the retina. Melanin-containing melanosomes are formed between early embryogenesis and up to 2 years in humans. Lipofuscin granules accumulate with increasing age and melanolipofuscin granules are a feature of aged RPE (Feeney, 1978; Boulton, 2014). When the filtered light reaches the retina, it initiates the visual process, which requires tremendous amount of energy. Reactive oxygen species (ROS) is thus generated as a by-product of active metabolism by mitochondria in photoreceptors (Wong-Riley, 2010). ROS triggers oxidative stress and subsequent retinal damage and is strongly implicated in retinal degeneration (Ray et al., 2012; Bellezza, 2018; Ozawa, 2020). RPE harbor a high concentration of cellular enzymatic antioxidants (Newsome et al., 1990; Blanks et al., 1992; Oliver and Newsome, 1992; Miceli et al., 1994; Tate et al., 1995; Feng et al., 2010; Murthy et al., 2014; Biswal et al., 2018; Sun et al., 2018; Zheng et al., 2022b), which should facilitate the alleviation of oxidative stress in RPE themselves as well as the outer retina. Melanosomes also have been demonstrated to have a potential antioxidant role in RPE (Burke et al., 2011).

#### 4.1.1 *MYO7A*-associated Usher syndrome 1B

*MYO7A* encodes myosin VIIA in photoreceptors and RPE. Mutations in *MYO7A* are associated with the most common Usher Syndrome type 1B characterized by congenital deafness and progressive retinal degeneration (Smith et al., 1994). *Myo7a*-null mice reveals the function of *Myo7a* in the apical localization of melanosomes and phagosomes via actin-based motor activity in RPE as well as the selective transport of opsins and other phototransduction proteins to the outer segments in photoreceptors (Lopes et al., 2011). Consequently, mutations in *MYO7A* disrupt the proper function of melanosomes for light absorption to protect the retina as well as the localization of phototransduction machineries to initiate vision, which lead to visual impairment and retinal damage in patients.

### 4.2 Visual cycle

Phototransduction is a visual process that converts light into electrical signals in photoreceptors. Photoreceptors contain a high concentration of visual pigment (i.e., opsin) at the outer segments (Ebrey and Koutalos, 2001). The type of opsin defines the photoreceptor subtype. Rod photoreceptors harbor rhodopsin. Cone photoreceptors can be further categorized into L- (long, 564 nm), M- (medium, 533 nm) or S- (short, 437 nm) type depending on the maximal spectral sensitivity of their opsins. Rod and cone opsins are present in the membranous discs of outer segments. Each opsin molecule is covalently bound to chromophore 11-*cis* retinal to become light-sensitive. Upon photon capture, 11-*cis* retinal is isomerized to all-*trans* form, which triggers a conformational change in opsins and initiates a cascade of biochemical events to initiate the phototransduction cascade. The photobleached pigment releases all-*trans* retinal into the disc bilayer. All-*trans* retinal cannot be processed by photoreceptors and thus is transported back to RPE for recycling and then returned to photoreceptors in the *cis* form as part of the visual cycle (Strauss, 2005; Palczewski and Kiser, 2020).

#### 4.2.1 *ABCA4*-associated Stargardt disease

ABCA4 is a member of the superfamily of ATP-binding cassette transporters primarily localized along the rim region of photoreceptor outer segment disc membranes (Allikmets et al., 1997; Azarian and Travis, 1997; Illing et al., 1997) but is also expressed by RPE (Lenis et al., 2018). Mutations in *ABCA4* are the major cause of Stargardt disease and a subset of cone-rod dystrophy with progressive blindness in children and young adults (Allikmets et al., 1997; Maugeri et al., 2000; Burke et al., 2014).

In photoreceptors, the free all-*trans* retinaldehyde combines rapidly and reversibly with phosphatidylethanolamine (PE) in the disc membrane to form *N*-retinylidene-phosphatidylethanolamine (*N*-ret-PE). While the retinylidene-bearing head group facing the outer segment cytoplasm is reduced to all-*trans*-retinol by retinol dehydrogenase 8 in the first step to regenerate visual chromophore (Rattner et al., 2000), the one located on the disc luminal surface is flipped to cytoplasmic side by ABCA4 (Sun and Nathans, 1997; Quazi et al., 2012). As retinaldehyde is toxic to photoreceptors (Getter et al., 2019), its efficient clearance and recycle not only facilitate the continuation of visual cycle but also maintain photoreceptor survival. Therefore, mutations in *ABCA4* lead to accumulation of retinaldehyde and delay the visual cycle, which contribute to visual impairment and photoreceptor degeneration in IRD. A key pathologic feature of Stargardt disease is the accumulation of fluorescent lipofuscin granules in RPE. As *ABCA4* has long been considered to be a photoreceptor-specific gene, the RPE phenotype is thought to be the major lipofuscin fluorophore A2E converted from bisretinoids from the outer segments with accumulation of retinaldehyde (Mata et al., 2000). However, *ABCA4* is found to be expressed by RPE in a recent study (Lenis et al., 2018). The RPE of dark-adapted *Abca4* mice accumulate lipofuscin the same rate as the ones under normal diurnal cycle, suggesting the lipofuscin is not contributed by the phagocytosed outer segments with accumulated retinaldehyde. Further investigation reveals that *ABCA4* recycles the retinaldehyde released from the phagocytosed photoreceptor outer segments in RPE endolysosomes (Lenis et al., 2018). RPE-specific expression of *ABCA4* show partial rescue of both the lipofuscin accumulation and photoreceptor degeneration (Lenis et al., 2018), suggesting that the phenotypes in *ABCA4*-Stargardt are contributed by both RPE and photoreceptor pathologies.

#### 4.2.2 *LRAT-, RPE65-, RLBP1*-associated RP

*LRAT*, *RPE6, and RLBP1* encode for enzymes involved in the visual cycle. Lecithin retinol acyltransferase encoded by *LRAT* catalyzes the first critical step to esterify all-*trans* retinol from photoreceptors into all-*trans* retinyl ester. Retinoid isomerohydrolase encoded by *RPE65* then converts the all-*trans *retinyl ester to 11-*cis* retinol, which is transported to the photoreceptors (4). Mutations in either of these two RPE-specific genes limit the availability of 11-*cis* retinal to photoreceptors, leading to early-onset visual impairment and subsequent photoreceptor degeneration. The transport of all-*trans* retinol from photoreceptors to RPE as well as 11-*cis* retinol from RPE to photoreceptor are carried by Retinaldehyde-binding protein 1 (RLBP1) (Xue et al., 2015; Napoli, 2016). Therefore, RLBP1 prevents the accumulation of toxic retinoid compounds in photoreceptors and RPE and facilitate the completion of visual cycle. Mutations of *RLBP1* could lead to early-onset visual impairment and retinal degeneration.

### 4.3 Metabolism

Photoreceptors and RPE share a unique symbiotic relationship in their co-dependent metabolic pathways. Each day, approximately 10% of photoreceptor outer segments are phagocytosed by RPE for daily renewal (Kevany and Palczewski, 2010; Viegas and Neuhauss, 2021). Phagocytosis of the outer segments also facilitates nutrient supply to photoreceptors. Glucose is transported from the choroidal circulation and supplied to photoreceptors by RPE through glucose transporter 1 (GLUT1), which is maintained at the apical side of RPE by phagocytosis of photoreceptor outer segments (Figure 2). Glucose is preferentially supplied to photoreceptors to maintain their high metabolic demand (Hurley, 2021). In photoreceptors, glucose is converted to ATP as energy source and, to lactate, which is shuttled to RPE for energy. Lactate is then converted to pyruvate by lactate dehydrogenase (LDH) in RPE to produce ATP through the Krebs or tricarboxylic acid (TCA) cycle and reduce NAD^+^ to NADH to inhibit glycolysis (Kanow et al., 2017; Hurley, 2021). Another approach to inhibit glycolysis in RPE is to activate the Akt pathway by phosphatidylserine on the outer segments (Viegas and Neuhauss, 2021). As photoreceptors are rich in lipids, the remaining products from phagocytosed outer segments contain sufficient phospholipids, fatty acids, cholesterol, and proteins to support the energy demand of RPE (Nolan et al., 2021; Ramachandra Rao and Fliesler, 2021; Viegas and Neuhauss, 2021). These lipids are broken down into ketone bodies by hydroxymethylglutaryl-coenzyme A (CoA) synthase 2 in RPE through the mitochondrial β-oxidation pathways (Nolan et al., 2021). The ketone bodies are released to the apical side of the RPE probably to be taken up by photoreceptors as another source of energy supply (Nolan et al., 2021). Approximately 80% of materials in the phagocytosed outer segments are recycled back to photoreceptors or removed to the blood stream as waste, a process regulated by ATP-driven Na^+^/K^+^ pumps (Mazzoni et al., 2014; Country, 2017; Kwon and Freeman, 2020).

#### 4.3.1 *MERTK*- and *TULP1*-associated RP

*MERTK* encodes a widely expressed receptor tyrosine kinase Mer, which is involved in numerous cellular processes and signal transduction pathways. The onset of visual impairment in *MERTK*-RP patients is within the second decade of life, with progressive decline of visual acuity (Gal et al., 2000; Tschernutter et al., 2006).

In the retina, MERTK is expressed in RPE and involved in the phagocytosis of outer segments of rod photoreceptors. Disruption of this process caused by *MERTK* mutations could impede the energy supply to RPE. Due to lack of lactate and reduced Akt activity, RPE starts to uptake glycolysis and starve photoreceptors.

Tubby like 1 (TULP1) binds to MERTK to stimulate RPE phagocytosis. Mutations of *TULP1* are also associated with severe early-onset IRD (Table 1). TULP1 is also expressed in photoreceptors, in which it is localized in the inner segments and engaged in the trafficking of photoreceptor opsins to the outer segments (Grossman et al., 2011; Palfi et al., 2020). The essential roles of TULP1 in both RPE and photoreceptors explain the more severe phenotypes in RP carrying *TULP1* mutations the *MERTK* ones.

#### 4.3.2 *PROM1*-and *VWA8*-associated IRD

Autophagy is a surveillance mechanism to degrade nucleic acids, lipids, and proteins to maintain cellular homeostasis. The autophagy pathway is responsible to breakdown the phagocytosed outer segments. Dysregulation of autophagy has been associated with various ocular disorder (Frost et al., 2014). Mutations in *PROM1* and *VWA1*, both of which are implicated in autophagy, could interfere with RPE metabolism and cause various types of IRD (Bhattacharya et al., 2017; Kong et al., 2023). *PROM1* encodes for Prominin-1 and is located to the open rims of photoreceptor outer segments to regulate disc morphogenesis in *Xenopus laevis* (Han et al., 2012; Carr et al., 2021). *PROM1*-IRD could be contributed by impaired disc formation. RPE-specific von Willebrand factor A domain containing 8 encoded by *VWA8* is well known for the regulation of mitophagy (i.e., autophagy of the mitochondria). Mutations in *VWA8* aberrantly activate the degradation of mitochondria and lead to defective retinal development and subsequent retinal degeneration in autosomal dominant RP (Kong et al., 2023). Surprisingly, treatment of malaria drug chloroquine or hydroxychloroquine, both of which act as autophagy inhibitor, could lead to damage to the macular cones outside of the fovea due to reduced lysosomal activity and outer segment phagocytosis (Stokkermans et al., 2023). Therefore, photoreceptor degeneration in *VWA8-*RP could be caused by compromised RPE metabolisms and/or retinal developmental defects.

#### 4.3.3 Bietti’s Crystalline Dystrophy

First described by Italian Ophthalmologist Dr. G.B. Bietti in 1937, Bietti’s Crystalline Dystrophy (BCD) is a rare autosomal recessive ocular disease characterized by yellow-white crystalline lipid deposits in the retina and sometimes cornea, degeneration of RPE, and sclerosis of the choroidal vessels (Saatci et al., 2023). The typical onset of BCD is between the second and third decades of life, and patients gradually lose peripheral and/or central visual acuity till legal blindness (Sayadi and Mekni, 2022). Although the pathophysiology of BCD is not yet fully understood, it is mainly caused by biallelic mutations in *CYP4V2* (Lin et al., 2005). *CYP4V2* encodes for a member of the cytochrome P450 hemethiolate protein superfamily which is involved in oxidizing fatty acid precursors. Dysfunctional lipid metabolism in RPE may disrupt the metabolic homeostasis between photoreceptors and RPE. Loss of fatty acid metabolism reduces the ketone bodies supplied to photoreceptors. RPE may consume glucose as energy supply, which disrupts photoreceptor function and leads to their starvation.

### 4.4 Ion channels

RPE express voltage- and ligand-gated potassium ion (K^+^), chloride ion (Cl^−^), and calcium ion (Ca^2+^)-conducting channels. These ion channels are crucial not only for the normal physiology of RPE but also for the interaction with photoreceptors. In the darkness, the K^+^ ions enter RPE through their Na^+^/K^+^-ATPases at the apical side and exit by the basolateral membrane to control the K^+^ concentration in the subretinal space and maintain the Na^+^-K^+^ equilibrium in photoreceptors (Baylor, 1996). When exposed to light, the hyperpolarization of photoreceptors reduces the release of K^+^ and leads to hyperpolarization of the apical membrane of RPE (Oakley, 1977), which subsequently inhibits the Na^+^/K^+^/2Cl^−^ co-transporters and results in an increase of Na^+^ and a decrease of the intracellular Cl^−^ concentration. Recent studies demonstrate that Na^+^ channels are strongly implicated in phagocytosis of photoreceptor outer segments and the lateral spread of voltage spikes via gap junctions in RPE (Johansson et al., 2019; Ignatova et al., 2023). Besides the Na^+^ channels, Cl^−^ and Ca^2+^ channels are also implicated in the phagocytosis of photoreceptor outer segments and their subsequent degradation by the autophagy pathway (Busschaert et al., 2017; Zheng et al., 2022a). Genetic mutations of chloride intracellular channel 4 (*CLIC4*) have been shown to lead to dry age-related macular degeneration potentially via dysregulation of the autophagy pathway, although the precise mechanisms require further investigation (Chuang et al., 2022). Cl^−^ channels, together with the K^+^ and Ca^2+^ ones, also have an important function in transepithelial transport of ions and water (Wollmann et al., 2006; Wimmers et al., 2007). The retina generates a large amount of water due to the high metabolic turnover. This water is eliminated by both RPE and MG. Transepithelial water transport from the apical to the basolateral side of the RPE is achieved by Ca^2+^-dependent modulation of K^+^ or Cl^−^ channels and aquaporin-1 channels (Stamer et al., 2003; Dvoriashyna et al., 2020).

#### 4.4.1 *KCNJ13*-LCA

*KCNJ13* encodes for potassium inwardly rectifying channel subfamily J member 13 in RPE. Mutations in *KCNJ13* lead to LCA16 characterized by significant central and peripheral vision loss in young children. Although the underlying mechanisms are not yet fully understood, mutations in *KCNJ13* lead to compromised cell alignment and phagocytosis in human induced pluripotent stem cell-derived RPE (Kanzaki et al., 2020), which was consistent with the function of K^+^ and relevant ions (e.g., Cl^−^, Ca^2+^) in the regulation of phagocytosis.

#### 4.4.2 Bestrophinopathy

Bestrophinopathy is the collective term of a phenotypically heterogeneous group of degenerative ocular diseases caused by mutations in the Bestrophin (*BEST*) genes, specifically the *BEST1* gene (Pasquay et al., 2015; Guziewicz et al., 2017). Initially *BEST* mutations were identified in IRD including Best vitelliform macular dystrophy (VMD), which is the most common form, autosomal dominant vitreoretinochoroidopathy (ADVIRC), and autosomal recessive bestrophinopathy (ARB). *BEST1* mutations are subsequently found to be implicated in more complex ocular disorders with the involvement of the anterior segment such as autosomal dominant microcornea, early-onset cataract, and posterior staphyloma (MRCS) syndrome (Johnson et al., 2017; Pfister et al., 2021). BEST1 is a Ca^2+^-activated Cl^−^ channel localized to the basolateral membrane of RPE (Marmorstein et al., 2000). Although the exact role of *BEST1* in RPE is unclear, its mutations cause a spectrum of phenotypes associated with compromised ion channels including altered permeability to large anions, dysregulated intracellular Ca^2+^ signaling, impaired anion channel activity, and mistrafficking of protein to the basalaterol membrane of RPE.

### 4.5 Immaturity of RPE in retinal ciliopathy

The primary cilium is a ubiquitous, microtubule-based organelle for modulating diverse signaling pathways and sensing external environment (Chen et al., 2021a). Mutations in genes associated with primary cilia are a major caused of IRD (Zelinger and Swaroop, 2018). Although primary cilia present in various retinal cell types (Lepanto et al., 2016; Ning et al., 2021), how defects of the primary cilia impact the function of every cell type is largely unexplored. *CEP290* encodes for a centrosomal/ciliary protein located at the transition zone and is responsible for initiating its formation by tethering the microtubules to the ciliary membrane (Craige et al., 2010; Wu et al., 2020). Mutations in *CEP290* compromise the formation of the transition zone and thus disrupt the biogenesis photoreceptor outer segments (Rachel et al., 2012; Rachel et al., 2015; Parfitt et al., 2016; Shimada et al., 2017). *CEP290*-LCA patients suffer from visual impairment at birth or infancy, with rod photoreceptors degenerating within the first decade of life followed by cone cell death (Cideciyan and Jacobson, 2019). A recent study indicates patient-derived RPE harbor defective apical processes, compromised phagocytosis, and reduced adult-specific gene expression (May-Simera et al., 2018). As the primary cilia is a regulator of various signaling pathway, the immaturity of RPE is caused by simultaneously suppressing canonical WNT and activating PKCδ pathways due to compromised primary cilia. Notably, such RPE defects precedes photoreceptor degeneration in a ciliopathy animal model (May-Simera et al., 2018). Ablation of primary cilia specifically in RPE also leads to photoreceptor degeneration in another animal model (Kretschmer et al., 2023). Therefore, although photoreceptor degeneration the major phenotypes in IRD caused by defective primary cilia, compromised RPE function could accelerate this process.

## 5 MG-photoreceptor interaction in IRD

Following injury or photoreceptor degeneration at the late stages of IRD, MG in the retina of fish and many non-mammalian vertebrates are able to dedifferentiate into neural progenitors which have the capacity to differentiate into all retinal neurons to replace the lost ones (Raymond et al., 2006; Bernardos et al., 2007; Thummel et al., 2008). However, MG in mammals have only limited capacity to regenerate the retina. Recent studies start to reveal growth factors and transcriptional machineries involved in the reprogramming of MG (Lahne et al., 2020; Todd et al., 2021; Todd et al., 2022). On the other hand, mammalian MG have adapted morphological, biochemical, physiological, and genetical machineries to activate reactive gliosis in response to the loss of retinal cells (Dyer and Cepko, 2000; Bringmann et al., 2009; Goldman, 2014; Vecino et al., 2016). Upon photoreceptor cell death, MG form scars to prevent further expansion of the injuries. Although this process is beneficial for photoreceptor survival, it may accelerate the degeneration process upon prolonged activation (Bringmann et al., 2006).

Indeed, MG are among the first cell types to respond to photoreceptor stress by secretion of antioxidants and neurotrophic factors (Jones et al., 2016; Leinonen et al., 2023). A recent study reveals MG can uptake damaged mitochondria from cone photoreceptors in zebrafish (Hutto et al., 2023). Therefore, compromised MG could contribute to photoreceptor degeneration during IRD progression. Consistently, MG-associated genes were found to be dysregulated at the early stages of IRD, which precedes photoreceptor dysfunction (Deng et al., 2018; Chen et al., 2023a). In this section, we will describe the role of MG in the retina and discuss how disease-causing genes impact MG function and lead to photoreceptor degeneration.

### 5.1 Structural support

**FIGURE 3:**
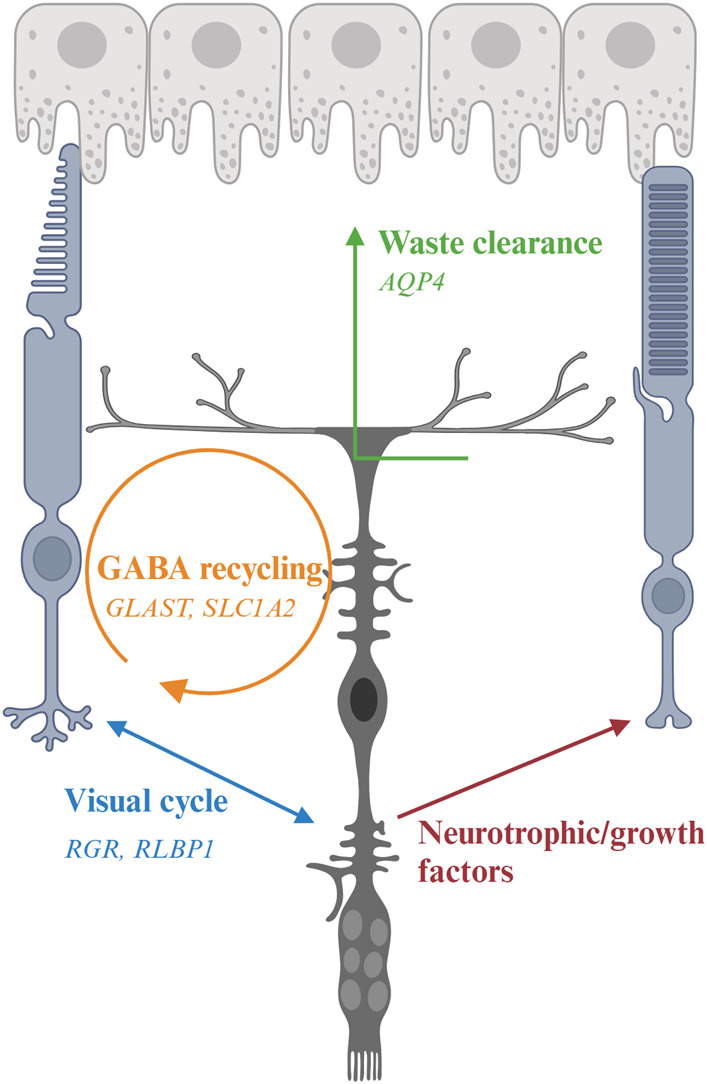
Interaction of Müller glia (MG) and photoreceptor and genes associated with inherited retinal degeneration. MG maintain osmotic homeostasis of the retina by AQP4 that facilitate the transport of ions and water. MG support PR by recycling glutamate and GABA neurotransmitters with the aid of other retinal cells, a process modulated by multiple genes including *GLAST*, and releasing neurotrophic factors. MG also facilitate the visual process of cone photoreceptors by expressing *RGR* and *RLBP1*.

MG are the predominant glial cells in the retina. The somata of MG reside in the INL and the two stem processes radiate in opposite directions, spanning the entire thickness of the retina (Figure 1). MG keep close contact with all types of retinal neurons to provide essential structural and functional support for their development and survival (Figure 3). In vertebrate retina, the apical processes of MG are attached to each other and to the inner segments of photoreceptors to form the outer limiting membrane (OLM) by adherens junctions and desmosomes. The adherens junctions interact with the actin cytoskeleton and intermediate filaments through zonula occludens (ZO)-1 and desmosomes respectively (Hartsock and Nelson, 2008). They also contain transmembrane proteins such as cadherins to interact with various cytoplasmic proteins (Chifflet et al., 2004). Adherens junctions perform multiple cellular functions including initiation and stabilization of cell–cell adhesion, regulation of the actin cytoskeleton, intracellular signaling, and transcriptional regulation (Omri et al., 2010). Mutations in OLM-associated genes interfere with photoreceptor maturation, function, and vision, and are implicated in multiple IRD including LCA, RP, and childhood cone-rod dystrophy as well as syndromic disorders (e.g., Usher syndrome) (Table 2) (Figure 3) (Khan et al., 2011; Bujakowska et al., 2012; Ratnam et al., 2013; Quinn et al., 2019b).

#### 5.1.1 *CRB1*-associated IRD

Mutations in the *CRB1* gene are associated with variable phenotypes in various IRD including LCA and RP (Bujakowska et al., 2012). Some patients also develop macular dystrophy (Bujakowska et al., 2012). The Crumbs (CRB) protein was first identified in *Drosophila* as a key regulator of apical polarity (Tepass et al., 1990). It is expressed in the retina and the brain. Among the three genes of the family in humans, *CRB1* and *CRB2* are expressed in the photoreceptor and MG (Pellissier et al., 2014; Quinn et al., 2019a). CRB1 contains transmembrane and cytoplasmic domains. It constitutes the adherens junctions and interacts with the actin cytoskeleton through the cytoplasmic domain (Gosens et al., 2008). Consistent with the function of adherens junctions, CRB1 has an evolutionarily conserved function to regulate cellular polarity. Animal models carrying *Crb1* mutations display disruption of the ONL, disorganization of the retinal layers, and loss of photoreceptor cell polarization (Mehalow et al., 2003; van de Pavert et al., 2004). The phenotypes of the animal models are consistent with the clinical features of patients carrying *CRB1* mutations, whose retinas are thickened and show an altered lamination (Jacobson et al., 2003), suggesting an important function of CRB1 in the formation of the ONL and the regulation of retinal morphogenesis. Although *CRB2* mutations in patients do not display phenotypes associated with the retina, *CRB2* has been shown to be a modifier of *CRB1* in diseases (Pellissier et al., 2014; Quinn et al., 2019b). MG-specific knockout of *CRB1* and knockdown of *CRB2* in mice and patient retinal organoids reveal disorganization of retinal structure and visual defects (Buck et al., 2021; Boon et al., 2023), suggesting that photoreceptor degeneration in *CRB1*-associated IRD is at least partially contributed by MG.

### 5.2 Clearance of ions, water, and cellular debris

The phototransduction cascade triggers hyperpolarization of photoreceptors by modulation of ion channels in their cell membranes. The visual signals are transmitted to second-order interneurons such as bipolar cells in the INL through neurotransmitters at the synaptic terminals. These processes are modulated by ions such as K^+^ and Na^+^, which affect conductance and permeability of the channels (Oakley, 1977; Mao et al., 2003). However, excessive K^+^ accumulated at the extracellular space has long been shown to lead to cell apoptosis (Bortner et al., 1997; Hughes and Cidlowski, 1999). MG mediate the transportation of excessive extracellular K^+^ to extraretinal fluid-filled space (blood vessels, vitreous, and subretinal space) via passive currents through Kir channels (Bringmann et al., 2006; Reichenbach and Bringmann, 2013; Reichenbach and Bringmann, 2020). Excessive water is also removed by MG via the aquaporin-4 (AQP4) channel (Reichenbach and Bringmann, 2013). Clearance of excessive water is critical to protect retinal neurons since water accumulation has been proposed as a pathogenic factor for retinal degeneration (Vecino et al., 2016). MG also phagocytose outer segments shed from cone photoreceptors as well as other cell debris to maintain retinal homeostasis (Bejarano-Escobar et al., 2017).

#### 5.2.1 *AQP4*-associated IRD

Aquaporin 4 encoded by *AQP4* is a membrane transport protein expressed in multiple epithelial and neurosupportive cells (Li et al., 2014). In the retina, AQP4 is highly expressed in MG and astrocytes. The expression and localization of AQP4 are dependent on syntrophins, and the elimination of α1-and β1-syntrophins induce an almost complete loss of AQP4 (Neely et al., 2001; Katoozi et al., 2020). Studies have shown that reduced AQP4 level causes altered MG cell volume (Netti et al., 2021). Depletion of AQP4 increases the susceptibility of MG toward osmotic stress and renders a higher risk of retinal degeneration upon light damage (Pannicke et al., 2010; Li et al., 2014). In *Aqp4* ^−/−^ mice, retinal hyperfusion and upregulated GFAP are observed, which consequently associated with the loss of retinal ganglion cells in congenital glaucoma (Maisam Afzali et al., 2022).

### 5.3 Regulation of synaptic transmission

Another well-studied function of MG is the regulation of synaptic transmission via recycling of neurotransmitter glutamate and gamma-aminobutyric acid (GABA). Glutamate is the most abundant excitatory neurotransmitter in the central nervous system including the retina (Zhou and Danbolt, 2014). MG uptake the released glutamate from excitatory retinal neurons by glutamate-aspartate transporter [GLAST; also known as excitatory amino acid transporter 1 (EAAT1) or solute carrier family 1, member 3 (SLC1A3)] to prevent ion toxicity and to maintain a fine visual resolution (Bringmann et al., 2006; Bringmann et al., 2013; Vandenberg and Ryan, 2013). The glutamate in MG is converted to glutamine by glutamine synthetase, an enzyme exclusively expressed by glia cells in retina (Pfeiffer et al., 2020). The glutamine is then recycled by retinal neurons for synthesis of glutamate and GABA. GABA is the main inhibitory neurotransmitter in retina (Yang, 2004). By recycling GABA via corresponding receptors and transporters, MG act as important modulators of visual processes through a fast termination of GABAergic signaling via their highly efficient GABA uptake (Biedermann et al., 2002).

#### 5.3.1 Animal models and patient data associated with *GLAST*

Although no IRD-causing mutations in glutamate-aspartate transporters have been reported, animal models and glaucoma patient samples reveal the role of GLAST in retinal degeneration. As glutamate transporters play a critical role in the recycling of glutamate, impaired function of glutamate transporters could cause glutamate accumulation in the extracellular matrix and contribute to excitotoxicity to retinal neuronal cells. Downregulation of GLAST expression has been reported in human glaucomatous eyes (Naskar et al., 2000) and mutations in *GLAST* are also found in glaucoma patients (Yanagisawa et al., 2020). Consistently, overexpression of *Glast* by AAV transduction protects retinal ganglion cells from degeneration in experimental autoimmune encephalomyelitis rats (Boccuni et al., 2023), highlighting a protective role of MG on photoreceptors.

#### 5.3.2 *CEP290*-LCA

Although MG harbor primary cilia, their function in MG remains largely unexplored. A recent study reveals dysregulation of gene associated with MG development and function in *CEP290*-LCA patient-derived retinal organoids (Chen et al., 2023a). Notably, the expression of *GLU1*, which encodes for glutamine synthetase to convert glutamine from glutamate, is downregulated in patient retinal organoids. However, how it impacts synaptic transmission is not investigated in this study. Whether the dysregulation of MG-associated genes is due to defects of the primary cilia caused by *CEP290* mutations or caused by the response of MG to photoreceptor dysfunction requires further investigation.

### 5.4 Secretion of neurotrophic and growth factors

MG secrete a variety of trophic and growth factors to regulate neuronal survival and neuritogenesis and to protect retinal neurons against excitotoxicity (Vecino et al., 2016; Tworig and Feller, 2021). Some well-studied examples include pigment epithelium-derived factor (PEDF), vascular endothelial growth factor (VEGF), glial cell-derived neurotrophic factor (GDNF), interleukin-6 (IL-6), ciliary neurotrophic factor (CNTF), brain-derived neurotrophic factor (BDNF), and nerve growth factor (NGF). Retinal ganglion cells and photoreceptors together with MG itself have receptors for these neurotrophins and growth factors.

#### 5.4.1 VEGF in IRD

Although VEGF is mainly associated with age-related macular degeneration (AMD), diabetic retinopathy (DR), and retinopathy of prematurity (ROP) (Hu et al., 2021a), VEGF levels have been found to be dysregulated in IRD (Salom et al., 2008). Intravitreal injection of VEGF in *rd1* mice show enhanced proliferation of retinal progenitor cells that have the potential to differentiate into retinal neurons (Nishiguchi et al., 2007). However, as there are no retinal progenitor cells even in newborn, the therapeutic potential of this approach remains to be determined.

### 5.5 Lipid metabolism

Photoreceptor outer segments are enriched in fatty acids and cholesterol, which are essential for maintaining metabolic homeostasis in the outer retina (see Section 4.3). These lipid components are channeled to photoreceptors by MG via their fatty acid-binding and transferring proteins. Docosahexaenoic acid (DHA), a trophic factor implicated in photoreceptor development and function, is taken up and processed by MG before supplying to photoreceptors (Politi et al., 2001; Shindou et al., 2017). MG also possess low-density lipoproteins (LDL) receptors that facilitate transport of circulating lipids to retinal neurons. This process is particularly crucial to maintaining photoreceptor outer segments and retinal ganglion cell axons as well as synapse formation (Mauch et al., 2001).

#### 5.5.1 APOE in IRD

Apolipoprotein E (APOE) is a plasma lipid transport protein that is mainly expressed in RPE but also expressed in MG and photoreceptors (Shanmugaratnam et al., 1997; Wickremasinghe et al., 2011; Hu et al., 2021b). APOE has been linked to the pathogenesis of AMD due to its function and potential role in drusen formation (Hu et al., 2021b). A recent study revealed dysregulation of APOE in *CEP290*-LCA patient-derived retinal organoids (Chen et al., 2023a). As these organoids harbor minimal RPE, the APOE is expressed by MG and/or photoreceptors, yet it is unclear which cell type(s) contribute to this phenotype. Other studies also indicate an association between APOE and glaucoma, but the association is controversial (Wang et al., 2014; Liuska et al., 2023). The role of APOE in IRD pathogenesis requires further elucidation.

### 5.6 Regulation of visual processes

Conversion of 11-*cis* retinal to the all-*trans* form in phototransduction requires the continuous recycling of the chromophore. Although RPE is the major site of this process in the visual cycle, the recycling rate is slow and the number of rod photoreceptors in human retina outweighs the cone ones, which may pose challenges for cone photoreceptors to obtain sufficient chromophore (Wang and Kefalov, 2011). Therefore, the presence of cone-specific visual cycle has long been proposed (Wang and Kefalov, 2011). MG express multiple retinoid-processing proteins such as CRALBP and retinol dehydrogenase-10 (RDH10), suggesting their implication in cone-specific visual cycle. Retinal G protein-coupled receptor (RGR) is a non-visual opsin in intracellular membranes of RPE and MG. It covalently binds to all*-trans* retinaldehyde and converts it to the 11-*cis* form (Hao and Fong, 1999). RGR lacks the motif to interact with G protein coupled receptor (Fritze et al., 2003), consistent with their role as a photoisomerase instead of a signaling molecule to activate the phototransduction cascade. Recent studies show that RGR couples with RDH10 to convert all-*trans* retinol to 11-*cis* retinol in a light-dependent manner (Morshedian et al., 2019; Tworak et al., 2023). Besides the role in cone-specific visual cycle, MG have also been reported to serve as optic fibers and direct the light to photoreceptors in guinea pigs (Agte et al., 2011), yet whether this function preserves in human retina remains further investigation.

#### 5.6.1 *RGR*- and *RLBP1*-associated IRD

Mutations in *RGR* is a pathogenic factor for RP (Table 2). Under continuous light treatment, cone photoreceptors of *Rgr* ^*−/−*^ mice lose their sensitivity sooner compared to the wild type ones (Morshedian et al., 2019). A recent study using an innovative cell type-specific gene reactivation approach confirms RGR is critical for cone photoreceptor function and such supporting function is contributed by both RPE and a subset of MG (Tworak et al., 2023). This finding raises an interesting yet challenging question on the targeted cell types for therapies of *RGR*-RP. Further investigation is also needed to identify the molecular signature of the MG subset responsible for the cone-specific visual cycle.

Likewise, mutations in *RLBP1* can cause various IRD including Bothnia dystrophy, retinitis pigmentosa, retinitis punctata albescens, fundus albipunctatus, and Newfoundland rod–cone dystrophy (Hipp et al., 2015) (Table 2). Compromised visual cycle and dysfunction of photoreceptors especially the cones are the primary phenotypes in patients (Kolesnikov et al., 2021). In *Rlbp1* ^−/−^ mice, reduced M-cone dark adaptation, mislocalization of opsin, and loss of photoreceptors are observed (Xue et al., 2015). These defects are demonstrated to be contributed by MG, and restoration CRALBP expression in MG improves M-cone sensitivity (Xue et al., 2015), suggesting an impact of MG pathology in visual defects of photoreceptors.

## 6 Recent advances in therapies

The advances in genetic approaches to identify targets, model systems to test therapeutics, and retinal imaging and molecular biology to evaluate therapeutic outcomes have established a promising environment for developing treatments for IRD. The first FDA approved gene therapy drug Luxturna further inspires the burgeon of numerous therapeutic approaches to maintain or restore vision. These proof-of-concept evidence not only demonstrates the clinical values but also provides new insights on the molecular mechanisms of disease pathogenesis. In this section, we will explore current therapeutic modalities to maintain photoreceptor survival and discuss how the role of RPE and MG in IRD pathogenesis could potentially impact the outcomes of IRD treatments. For therapeutic approaches to restore vision for late-stage IRD patients with little or no photoreceptors viable in the retina, we direct our readers to other excellent reviews discussing cell replacement therapy, retinal prosthetics, and direct brain stimulation (Yue et al., 2016; Bosking et al., 2017; Uyama et al., 2021; Chen et al., 2023b; Chew and Iannaccone, 2023).

### 6.1 Gene therapy

Gene-based therapy involves the delivery of genetic materials as an intervention to modify the expression of disease-associated proteins in target cells/tissues. The main goal of gene therapy is to restore the protein function compromised by the mutated genes permanently to reduce the need for long-term medication dependence (Mendell et al., 2021). Dating back to over seven decades to the first observation of viral gene transfer, gene therapy has been demonstrated to be a relatively safe and long-term efficacious therapeutic approach for treatment of various genetic disorders that once considered incurable (Wirth et al., 2013; Tamura and Toda, 2020). Although adverse side effects were reported in two human clinical trials in late 1990s (Wirth et al., 2013; Tamura and Toda, 2020), with the development of new technologies and viral vectors, gene-based therapies have been applied in over 200 human clinical trials involving various tissues and cell types without any incidents of deaths or cancers (Ginn et al., 2018).

As an enclosed, immune-privileged site protected by the blood-retina barrier (Chen et al., 2019), the retina offers a unique opportunity for gene therapy. Only low doses of gene therapy vectors are needed to treat the retina due to its small size and a lack of cellular proliferation in adulthood (Trapani and Auricchio, 2018), and thus the risk of systemic dissemination of the vectors and immune responses is generally negligible (Amato et al., 2021). In addition, surgical procedures or clinical practices have been well established to deliver the gene therapy machineries into the retina, and the therapeutic outcome can be easily monitored by ocular imaging technologies including optical coherence tomography and fundus imaging (Drag et al., 2023). Therefore, gene therapy has been extensively evaluated in the retina in the past two decades. Currently, 73 clinical trials for IRD are recruiting, 39 starting soon, and 154 have been completed (https://clinicaltrials.gov/). Disorders being targeted for genetic therapy include RP, LCA, choroideremia, achromatopsia, Leber’s hereditary optic neuropathy, usher syndrome, X-linked retinoschisis, and Stargardt disease (Nuzbrokh et al., 2021). Besides IRD, gene therapy has also been applied for treatment of age-related macular degeneration by inhibiting VEGF (Heier et al., 2017; Grishanin et al., 2019) or the complement cascade (Cashman et al., 2011).

#### 6.1.1 Current progress and clinical success

##### 6.1.1.1 Strategies of gene-based therapy

Depending on the molecular mechanisms of disease pathogenesis, different strategies are applied in IRD treatment. When a disease-causing mutation disrupts the normal gene function, such as in the case of autosomal recessive or X-linked recessive IRD, gene augmentation (also called gene replacement) could be a promising approach to restore the normal function of the mutated gene. As one of the most commonly used gene therapy strategies, the feasibility and success of gene augmentation therapy in IRD treatments have been demonstrated by numerous clinical and preclinical studies. Besides *RPE65* expression by Luxturna for treatment of LCA, delivery of *ND4*, *REP1*, *ABCA4*, *RPGR*, *MERTK*, *RS1*, and *CNGA* are currently at various stages of clinical trials targeting Leber hereditary optic neuropathy (Yang et al., 2016; Guy et al., 2017; Zhang et al., 2019; Yuan et al., 2020), choroideremia (Dimopoulos et al., 2018; Lam et al., 2019; Morgan et al., 2022), Stargardt disease (Parker et al., 2022), X-linked RP (Cehajic-Kapetanovic et al., 2020), autosomal recessive RP (Ghazi et al., 2016), X-linked retinoschisis (Mishra et al., 2021), and achromatopsia (Fischer et al., 2020), respectively. Despite signs of inflammation and adverse outcomes in some cases, majority of the clinical studies demonstrate safety and/or efficacy of this approach in functional improvement of vision in patients. Besides delivery of normal genes, for IRD caused by splicing defects, antisense oligonucleotides (AON) have been shown to be a long-lasting and effective approach to restore normal splicing and consequently protein function, such as in the case of LCA caused by *CEP290* mutations (Cideciyan et al., 2019; Russell et al., 2022). A preclinical study of the AON approach also showed promising results for correcting the splicing defects in Usher syndrome caused by *USH2A* mutations (Slijkerman et al., 2016). CRISPR/Cas9-mediated genome editing has recently been successfully applied for treatment of *CEP290*-LCA in degenerative models, demonstrating safety and efficacy in the retina (Ruan et al., 2017). The newly developed self-limiting CRISPR/Cas9 system minimizes the duration of Cas9 expression to futher reduce the immune response (Ruan et al., 2017).

In contrast to loss-of-function mutations in recessive IRD, gain-of-function mutations in autosomal dominant diseases lead to the formation of aberrant proteins that disrupt normal cellular or tissue functions. In this case, the therapeutic goal is to prevent the expression of the altered genes. Downregulation of gene expression by ribozyme, small interfering RNA, and AON has been developed. Although these techniques have been well optimized and extensively applied in *in vitro* models, their application for therapeutics *in vivo* is still challenging due to the specificity, off-target effect, and degradation of RNA molecules. Currently, these transcriptional silencing strategies have been successfully applied in animal models of RP caused by *RHO* mutations (Lewin et al., 1998; Chadderton et al., 2009; Hernan et al., 2011; O'Reilly et al., 2008; Murray et al., 2015). A CRISPR/Cas9-mediated transcript degradation approach has also been successfully applied *in vitro* and preclinical models of *RHO*-RP (Bakondi et al., 2016; Li et al., 2018; Tsai et al., 2018). Besides autosomal dominant diseases, the genome-editing approaches, either by CRISPR/Cas9-system or by transcription activator-like effector nuclease (TALEN), can also be applied to correct the mutations underlying recessive disorders. One successful example is the correction of *Crb1* mutation in *rd8* degenerative model by TALEN-Mediated homology-directed repair. Even the heterozygous mice restore the morphology of the ONL and show a normal retinal phenotype (Low et al., 2014), suggesting the efficiency of the correction could meet the therapeutic needs.

##### 6.1.1.2 Delivery approach

Recombinant adeno-associated virus (AAV) vectors are currently widely used in ocular gene therapy due to therapeutic benefits including non-integrating nature, low immune response, and long duration of transgene expression (Wu et al., 2006; Amato et al., 2021). Recombinant AAV (rAAV) vectors offer a high number of tissue-specific serotypes including AAV 1, 2, 4, 5, 6, 7, 8, and 9 for retinal cells, and thus improve the specificity of viral transduction. The safety and efficacy of AAV-mediated gene therapy have been demonstrated by most of the completed and ongoing clinical studies including Luxturna. One notable limitation of AAV is the small packaging limit, and thus the genetic materials carried by AAV cannot exceed 4.7 kb (Wu et al., 2010), and therefore it is not applicable for diseases caused by mutations of large genes. Lentiviruses, a retrovirus with a larger packing capacity of up to 8kb, become a more compelling alternative to AAV vectors in this case. Lentiviruses pose risk of mutagenesis due to their nature to integrate into the host genome, yet such risk could be justified for treatment of the post-mitotic retina (Drag et al., 2023). The initial trial of *ABCA4* delivered by lentiviral vectors for treatment of Stargardt disease has showed promising safety data and the evaluation of efficacy is currently ongoing (Parker et al., 2022).

Despite the positive data from lentiviral vectors, they still pose risks for insertional mutagenesis, germline transmission, and adverse immune response (Drag et al., 2023). Non-viral delivery of genetic materials is being developed as an alternative for IRD caused by mutations of large genes. Although non-viral delivery by physical (e.g., direct injection of genetic materials) or chemical (e.g., nanoparticles) approaches are not as popular as the viral ones due to the low efficiency, immune response, and duration of the effect, recent advances have started to overcome these issues (Sharma and Paschalis, 2022). Various bioengineering materials such as synthetic polymers, lipid cations, liposome-based nanoparticles, and polysaccharides show good transfection efficiency. The use of polyethylene glycol (PEG) combined with cell type-specific promoter or ligand could largely improve the specificity and efficacy of therapies and reduce immunogenicity. The feasibility of such approach has been demonstrated by the relatively efficient and durable therapeutic outcomes in cell cultures and an *ABCA4*-associated animal model in a recent study (Sun et al., 2020).

##### 6.1.1.3 Administration route

Gene therapies are typically delivered to target retinal cells by subretinal or intravitreal injection in clinical studies. Subretinal injection is a commonly used approach to deliver gene therapy machineries to the subretinal area for treatment of RPE and/or photoreceptors (Planul and Dalkara, 2017). In addition, the subretinal area is a closed immune-privileged compartment and thus further reduce the immune response (Xian and Huang, 2015; Chen et al., 2019). However, subretinal injection could potentially trigger several complications including retinal tears, cataract progression, retinal detachment, or retinal/choroidal hemorrhages (Tripepi et al., 2023). Such a delicate procedure also requires skilled and experienced surgeons for successful administration (Huang et al., 2022).

Intravitreal injection offers several advantages over subretinal injection. It is less invasive and less technically challenging, thus can be performed in a clinic setting and offer the opportunity of gene therapy to larger populations (Amato et al., 2021). However, although efficiently transducing inner retinal cells, intravitreal injection is less effective on outer retinal cells due to dilution of vectors in vitreous cavity and the thick inner limiting membrane in primates (Planul and Dalkara, 2017). A higher therapeutic dose of vectors is thus needed to achieve a desirable outcome, which could pose a significant risk of immunogenicity (Drag et al., 2023).

Suprachoroidal delivery in the space between the sclera and the choroid has also been shown to be a safe approach in preclinical and clinical studies yet it poses risk to spread viral vectors into the systemic circulation and could lead to adverse physical and immune response (Kansara et al., 2020; Naftali Ben Haim and Moisseiev, 2021; Tan et al., 2021).

#### 6.1.2 Strength and limitation

The success of gene therapy has been well demonstrated by the approval of FDA as the first therapy to treat IRD. Numerous of completed and ongoing clinical trials targeting IRD caused by various disease-causing genes show promising safety and efficacy data so far. Approvals of more gene-based therapies are expected in the near future. The advances in engineered viral vectors and cell type-specific promoters further improve the specificity and safety of this approach.

However, gene therapy still faces several problems waiting to be addressed. One of the biggest challenges of traditional gene therapy is their dependence on the targeted disease-causing genes. With over 280 disease-causing genes of IRD (RetNet, https://sph.uth.edu/retnet/) currently identified, it could be time-consuming, expensive, and labor-intensive to design gene therapy for individual gene. In addition, the gene(s) responsible for disease phenotypes are not always identified in patients. Next-generation whole exome sequencing in combination with genetic linkage or homozygosity mapping approaches should facilitate the identification of the causal genes and the design of effective gene therapy (Siemiatkowska et al., 2014). Different mutations in the same gene could lead to various clinical phenotypes, and mutations in different genes may show comparable clinical manifestations. Such complexity poses challenges to gene therapy targeting a single disease-causing gene. Besides, as a rare disease, developing treatments for each IRD may not be favorable for pharmaceutical companies. Therefore, gene-agnostic gene therapy independent of the mutated genes are being developed. This approach targets the converging pathway(s) in multiple IRD and thus has the potential to significantly reduce the cost of development and commercialization of IRD treatments. OCU400, a nuclear hormone receptor-based gene therapy, is currently under clinical trials for treatment of RP caused by *NR2E3* or *RHO* mutations (NCT05203939). AAV-mediated expression of *Txnip*, which modulates the nutrient supply, has been shown to prolong the survival of cone photoreceptors and improve visual acuity in multiple RP models (Xue et al., 2021), providing promising proof-of-concept evidence for the feasibility of this approach.

Another limitation of AAV-mediated gene therapy is the incapacity to delivery large genes. Although lentiviral vectors and non-viral approaches are being developed to carry large genes into the retina, they may lead to adverse immune response and safety issues and thus require further improvement. Concurrently, AAV-mediated dual vector approach, in which the disease-causing gene is split into two parts and packaged into separate AAV vectors, are being developed. Although the reconstitution efficiency was relatively low initially (McClements and MacLaren, 2017), a recent study improves the technology and demonstrates promising results in animal models and human pluripotent stem cell-derived retinal organoids (Riedmayr et al., 2023), suggesting the feasibility of this approach.

Besides, several clinical studies demonstrated confounding therapeutic outcomes, which may due to the treatment window and patient variability (Ghazi et al., 2016; Fischer et al., 2020; Mishra et al., 2021; Hahn et al., 2022). To maximize the therapeutic potential of gene therapy, a better understanding of disease pathogenesis and careful selection and characterization of patients during the recruitment period could be essential.

#### 6.1.3 RPE and MG as therapeutic targets

The clinical phenotypes of IRD caused by RPE-associated genes reveal a role of RPE in photoreceptor degeneration. Gene augmentation therapies by AAV vector carrying these genes driven by RPE-associated or CMV promoters have shown positive therapeutic outcome in preclinical and clinical studies (Choi et al., 2015; Ghazi et al., 2016; MacLachlan et al., 2018; Dyka et al., 2019; Sun et al., 2020). In the treatment of *CEP290*-LCA, although AON drug QR110 has shown favorable therapeutic outcome (Jacobson et al., 2017; Cideciyan et al., 2019), the AON is driven by CMV promoter that is able to drive gene expression in RPE and delivered by AAV2, which can readily transduce both photoreceptors and RPE. Therefore, whether the therapeutic outcome of QR110 is due to correction of CEP290 splicing defects in photoreceptor only or both photoreceptor and RPE requires further investigation. Mutations in genes associated with primary cilia are a major caused of IRD (Zelinger and Swaroop, 2018). Further investigation on RPE structure and function in IRD caused by mutations of ciliary genes is needed to determine whether RPE should be included as a therapeutic target in gene therapy.

Mutations in MG-associated genes including *RLBP1* and *CRB1* could lead to photoreceptor degeneration in IRD. Although gene augmentation therapies of *RLBP1* reveal visual improvement in preclinical models (Choi et al., 2015; MacLachlan et al., 2018), CMV promoter is applied to drive the expression of RLBP1 and thus it is difficult to dissect whether the therapeutic outcome is resulted from the improvement of RPE, MG, or both. Studies on *CRB1*- and *CRB2-* associated RP and LCA illustrate the role of MG in disease pathogenesis. Novel MG-specific RP mouse and human pluripotent stem cell-derived retinal organoid models, which harbor completely loss of CRB1 and reduced levels of CRB2 specifically in MG, reveal disorganization of retinal structure and visual defects (Buck et al., 2021; Boon et al., 2023). Delivery of CMV-*CRB2* is more potent than CMV-*CRB1* in protection of vision in animal models (Buck et al., 2021). Gene augmentation therapy with *CRB2* also improves the outer retinal phenotypes in *CRB1* knockout retinal organoids (Boon et al., 2023). These results suggest that compromised CRB1 and CRB2 functions in MG are the major cause of photoreceptor degeneration. In addition, a recent study indicates dysregulation of MG-associated genes involved in development and function in retinal organoids derived from *CEP290*-LCA patients (Chen et al., 2023a). Although the primary cilia are a ubiquitous organelle, their roles are not heavily investigated except in photoreceptors. Therefore, it is unclear whether the dysregulated MG genes are caused by defects of the primary cilia or responses to compromised photoreceptors.

Understanding the cellular mechanisms of IRD pathogenesis is crucial for the success of a safe, long-lasting, and efficacious gene therapy. Current gene therapies have careful selection of AAV serotypes and cell type-specific promoters to limit the non-specific expression of the delivered genes. However, if mutations of the disease-associated genes compromise the function of RPE and/or MG, which contribute to pathogenesis, RPE and/or MG should be included as the targeted cell types. Transduction of RPE and MG in treatments will impact not only the selection of AAV serotype and promoter but also the route of administration as intravitreal injection is more efficient than subretinal injection for transducing inner retinal cells. Such as in the case of *RGR*-RP, the RGR-dependent cone visual cycle is mediated by both RPE and a subtype of MG (Tworak et al., 2023). It could be challenging to deliver viral vectors to both cell types efficiently. In addition, the dose of AAV should also be taken into account as RPE and MG are different cell types from photoreceptors and thus they may have distinct tolerance of AAV transduction and ectopic expression of genetic materials.

### 6.2 Pharmaceutical therapy

Although drug screening using cell lines have successfully identified therapeutics for various diseases, its application for IRD treatment remains slow due to the limited cell number in the retina and the technical challenge to maintain retinal primary cultures. Recent advances in synthetic chemistry, AI-based screening, structural biology, and the advances in pluripotent stem cell differentiation and high-throughput screening technologies should spur the design of novel bioactive molecules and drug repurposing for IRD treatment.

#### 6.2.1 Current progress

Antioxidants such as vitamin A, vitamin B3, DHA, and lutein have been shown to delay or inhibit the apoptosis of photoreceptors and preserve patient vision (Guadagni et al., 2015). Likewise, applications of neurotrophic agents including CNTF, BDNF, and anti-apoptotic drugs (e.g., tauroursodeoxycholic acid, rasagiline, norgestrel, and myriocin) have shown positive therapeutic outcomes in cell cultures and animal models of RP (Dias et al., 2018).

Besides inhibiting photoreceptor apoptosis to preserve vision, another approach is to target the compromised function and/or restore the dysregulated pathways associated with the mutated genes. A repurposed drug metformin has been shown to facilitate the clearance of lipid deposits caused by *ABCA4* mutations in human pluripotent stem cell-derived and *in vivo* RPE (Amin et al., 2022; Farnoodian et al., 2022). A selective estrogen receptor modulator raloxifene is also shown to inhibit photoreceptor apoptosis and mitigate toxicity of retinaldehyde in *ABCA4*-associated models (Getter et al., 2019). As Metformin is an FDA-approved treatment for type 2 diabetes, National Eye Institute is launching a Phase I/II trial to evaluate the therapeutic effect of Metformin on Stargardt disease patients. Lumacaftor, another FDA-approved drug for the treatment of cystic fibrosis, has been shown to rescue the *ABCA4* trafficking mutants by restoration of Hsp27 in HEK cells (Liu et al., 2019). Yet the therapeutic value of Lumacaftor for preserving vision requires more tests on degenerative models and/or human pluripotent stem cell-derived RPE. A flavonoid drug eupatilin is able to partially restore photoreceptor outer segments and visual function in degenerative models caused by *CEP290* mutations (Kim et al., 2018). Disruption of protein homeostasis is another common cause of photoreceptor degeneration in IRD. Modulations of this process, either by regulation of the autophagy pathway and the ubiquitin-proteasome system, or by the use of small molecule chaperon, is able to reduce photoreceptor apoptosis and preserve light detection ability in multiple degenerative models (Mockel et al., 2012; Chen et al., 2018; Yao et al., 2018; Qiu et al., 2019; Intartaglia et al., 2022; Chen et al., 2023a).

#### 6.2.2 Strength and limitation

Pharmaceutical drugs can be produced at reasonable costs and their manufacturing is scalable (Tambuyzer et al., 2020), which are desirable particularly for rare diseases like IRD. As pharmaceutical drugs act by targeting dysregulated cellular function and/or signaling pathways, which could be shared by multiple IRD caused by different mutations, they hold the promise to be translated into cost-effective gene-agonistic therapies. Various drugs currently identified for treatment of IRD act as neurotrophic factors or apoptotic inhibitors, and thus they could be applied together with gene therapy drugs to achieve better therapeutic outcomes. The establishment of high-throughput screening platform using retina of degenerative models (Campbell et al., 2018; Chen et al., 2018), cell lines (Kim et al., 2018), or pluripotent stem cell-derived retinal organoids (Chen et al., 2023a) should accelerate drug discovery for treatment of IRD.

However, pharmaceutical drugs, particularly the small molecules, are highly penetrant and thus could lead to off-target effects. Therefore, it is crucial yet challenging to identify the right molecule with an excellent pharmacological effect and pharmacokinetics and few off-target impact, which sometimes requires extensive optimization of the lead compound (Tambuyzer et al., 2020). A deeper understanding of the key signaling pathways involved in the therapeutic processes should facilitate the refinement of the lead compound. Furthermore, over 90% of drug candidates fail in Phase I clinical trials due to the differences in pathophysiology and pharmacokinetics between humans and animal models (Horvath et al., 2016). Human pluripotent stem cell-derived retinal organoids structurally and functionally recapitulate *in vivo* retina in various important aspect (Cowan et al., 2020; Saha et al., 2022) and thus offers a promising platform to test drugs in a human context. Recent studies have begun to employ the organoid platform to evaluate the dose and therapeutic outcome of drugs (Chen et al., 2023a; Corral-Serrano et al., 2023).

#### 6.2.3 Targeting RPE and MG by pharmaceutical approaches

Due to the penetrant property of pharmaceutical drugs, all retinal cell types including RPE and MG are targeted in the treatment. A recent study revealed partial restoration of dysregulated genes in patient-derived retinal organoids upon treatment of IRD drug reserpine (Chen et al., 2023a). However, it is unclear whether such improvement in MG is a therapeutic outcome of the drug treatment or the secondary effect of photoreceptor restoration. Furthermore, the therapeutic and toxic doses may differ among retinal cell types. Evaluation of all retinal cell types in the drug treatment should facilitate the selection of optimal doses to achieve desirable therapeutic outcomes without interfering the homeostasis of the retina. Besides, all targeted cell types should be included in the initial screening to avoid toxic side effects due to undesirable interaction of the drug with cell type-specific molecules. One typical example is the interaction of chloroquine and hydroxychloroquine with melanin, which abrogates the lysosomal activity of RPE and leads to subsequent macular degeneration (Stokkermans et al., 2023).

## 7 Conclusion and outlook

IRD is a common cause of childhood visual impairment and blindness, yet the disease mechanisms have not been comprehensively investigated and limited treatment options are currently available. A vast majority of IRD are caused by dysfunction and/or degeneration of photoreceptors. In healthy retina, photoreceptor homeostasis is maintained by MG and RPE, which have been shown to remove cellular debris, damaged mitochondria, and excessive ions and release trophic and growth factors. Photoreceptors on the other hand provide metabolites to MG and RPE as energy source. Therefore, photoreceptors tightly interact with MG and RPE and they share a symbiotic relationship to maintain homeostasis of the outer retina. Consequently, genetic mutations that compromise the one could disrupt the others. In favor of this postulation, recent studies reveal MG and RPE pathologies in IRD caused by mutations in photoreceptor-specific or ubiquitously expressed genes, suggesting photoreceptor degeneration could be contributed by cellular dysfunction resulted from genetic mutations and MG/RPE pathologies. MG and RPE should therefore be taken into account in designing treatments for IRD.

With over 280 IRD-causing genes, it is time-consuming and labor-intensive to develop gene therapy targeting individual disease-causing gene. Gene therapeutic approaches targeting shared dysregulated functions (e.g., immune response, glucose metabolism, oxidative stress) among IRD caused by different mutations are being developed (Venkatesh et al., 2015; Xiong et al., 2015; Wang et al., 2019; Xue et al., 2021). MG/RPE pathologies in IRD could be at least partially contributed by their response to photoreceptor dysfunction such as phagocytosis of cell debris and damaged mitochondria. Therefore, designing therapeutics targeting relevant compromised MG/RPE function should hold the promise to be developed into mutation/gene-independent treatments targeting multiple IRD.
